# Voluntary and involuntary motor behaviours in the varieties of religious experience

**DOI:** 10.1093/braincomms/fcae471

**Published:** 2025-01-13

**Authors:** Christos Ganos, Michael A Ferguson, Kurt Gray, Andrew J Lees, Kailash P Bhatia, Patrick Haggard

**Affiliations:** Movement Disorder Clinic, Edmond J. Safra Program in Parkinson’s Disease, Division of Neurology University of Toronto, Toronto Western Hospital, Toronto, Canada M5T 2S8; Department of Neurology, Charité - Universitätsmedizin Berlin, Corporate Member of Freie Universität Berlin and Humboldt-Universität zu Berlin, 10117 Berlin, Germany; Center for Brain Circuit Therapeutics, Brigham & Women’s Hospital, Boston, MA 02115, USA; Department of Neurology, Harvard Medical School, Boston, MA 02115, USA; Department of Psychology and Neuroscience, University of North Carolina, Chapel Hill, NC 27599-3270, USA; Reta Lila Weston Institute of Neurological Studies, UCL, Institute of Neurology, London WC1N 1PJ, UK; Department of Clinical and Movement Neurosciences, UCL, Queen Square, Institute of Neurology, University College London, London WC1N 3BG, UK; Institute of Cognitive Neuroscience, University College London, London WC1N 3AZ, UK

**Keywords:** religious motor behaviours, volition, motor rituals, sense of agency, functional movement disorders

## Abstract

Religion is a widespread feature of human life. Religions typically include both distinctive varieties of experience and also a set of foundational beliefs. An additional, but often overlooked, part of many religions is their expression through specific actions, which we here designate religious motor behaviours. Here we describe these religious motor behaviours and offer a taxonomy based on the conceptual schemes of movement neuroscience and neurology. Thus, religious rituals include both behaviours characterized by decreased motor output (e.g. ritualistic silence) and behaviours characterized by increased motor output (e.g. ritual dances). Neurology often also distinguishes between movements that are *experienced* as voluntary or involuntary. We show that this same distinction can also apply to religious experiences, since these may be characterized either by a heightened sense of personal control or a sense of being controlled by an external, divine source. We then use these conceptual structures of movement neuroscience to investigate examples from a wide range of religious contexts. We thereby categorize religious motor behaviours into different classes, focusing on specific examples: repetitive ritual actions; motor behaviours where the experience of volition is altered, such as automatisms; and possession-like states. We suggest that a scientific approach to these behaviours should include their predominant phenomenological presentation, the accompanying subjective experience of volition and the underlying neurocognitive mechanisms. This investigation shows rich parallels between religious motor behaviours and motor behaviours observed in neurological disorders, including those that present with functional neurological symptoms. Our approach does not and should not pathologize religious motor behaviours, but rather draws attention to a rich set of non-clinical motor phenomena that highlights important social, cultural and psychological elements of human movement control. Movement neuroscience and religious activity have unexplored overlaps and can usefully learn from each other.

## Teaser

Ganos *et al*. present a taxonomy of ‘religious motor behaviours’, integrating concepts from movement disorders and cognitive neuroscience. This framework reveals several parallels between voluntary and involuntary religious actions and clinical motor phenomena, suggesting common neurocognitive pathways shaped by cultural contexts.

## Graphical Abstract

**Graphical Abstract:**
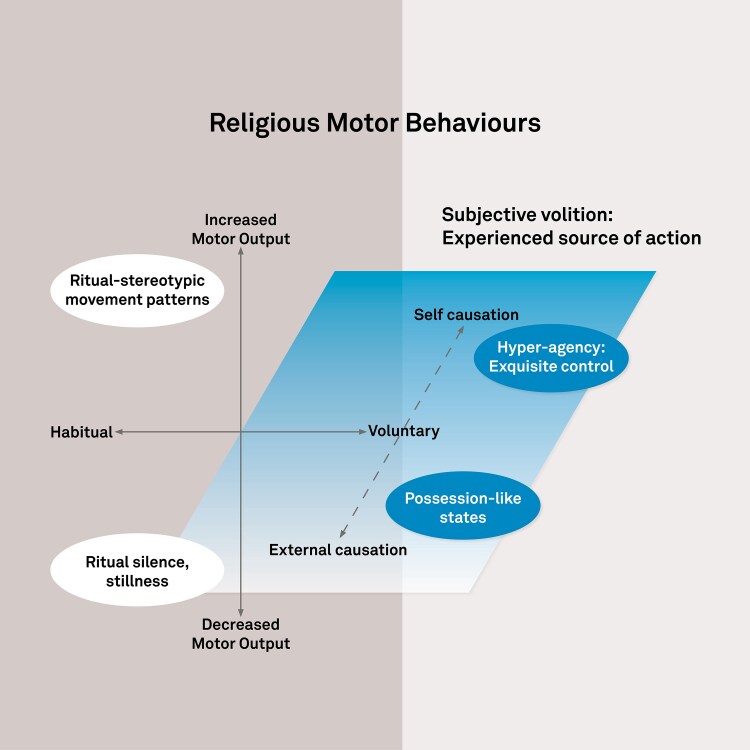

The field of movement disorders encompasses a wide spectrum of phenomena that either directly disrupt willed actions or lead to additional unwilled actions. A common feature, therefore, of all movement disorders is that they involve at least some level of involuntariness: there is either movement or lack of movement that lies outside the normal fluent voluntary control people have of their bodily actions. Clinical neurology routinely addresses both the reduction of spontaneous and willed action (e.g. parkinsonism) and also excessive, unwilled motor output (e.g. chorea, dystonia, myoclonus, tremor and tics). This primary, yet often unacknowledged, focus on regulating the ‘amount’ of movement that corresponds to a ‘normal’ level brings into focus an interesting additional set of cultural motor behaviours, which are also often considered different to ‘normal’, but are generally not considered from a clinical perspective.

Here we focus on the striking example of motor phenomena associated with different varieties of religious experience. To our knowledge, no previous studies have attempted to describe religious motor behaviours through the lens of movement disorders. Our goal in so doing is not to place these behaviours under a pathologizing medical lens. Furthermore, we do not aim to evaluate religion in general, nor make comparisons between religions. Instead, we aim to draw attention to a neglected class of human motor behaviours and to provide a novel view of relation between motor action and inner mental life.

The question of how ‘religious motor behaviours’ should be understood scientifically raises questions about the purpose and meaning of human voluntary actions. We make a major distinction between the voluntary motor behaviours that occur during religious practices, for example as part of praying, but do not involve altered ‘experiences’ of action, and other religious motor behaviours that apparently involve altered experiences of action, and for which this alteration often appears central to the religious aspect of the experience. We argue that there are overlooked parallels between the conceptual organization of movement disorders and the varieties of motor behaviours associated with religious experience. Understanding these parallels could be important for both fields. Furthermore, the strong parallels suggest that some movement disorders, particularly those functional disorders associated with altered experiences of volition, and some religious motor behaviours could share common underlying neural mechanisms.

There is a large body of literature on religious rituals and religious experiences.^1,2^ In his Gifford Lectures on Natural Religion, William James provided a remarkable ‘study in human nature surrounding the varieties of religious experience’.^3^ James defined religion as the domain of human life related to ‘the feelings, acts, and experiences of individuals as they apprehend themselves to stand in relation to whatever they may consider the divine’.^3^ In this treatise, he not only offered a valuable scientific taxonomy of religious phenomena but also provided key insights into the psychological traits that might drive some of the experiences and behaviours associated with them.

James recognized that a key effect of religious experience on believers was to influence their motor action. There is a wide variety of motor phenomena associated with religious practice. On the one hand, there are routine behaviours that occur as part of religious rituals. These may be completely voluntary behaviours (e.g. lighting a candle) but may also be stereotypic and almost habitual (e.g. body swaying during prayers, mudras, signs of the cross). Importantly, some of these behaviours are performed with an intention of ‘gathering the mind’, so as to better engage with ritualized stillness and silence. These routinely include, for example, repetitious finger motions for manipulating prayer beads, repetitious speech (such as a repetitively recited prayer or mantra), kneeling and prostration, and also the practice of simply remaining still until there is a movement (or speech) that, from a religious point of view, seems important to express—as in Quaker practice. Moreover, some ‘ritual stillness’ activities may appear to be hypokinetic at a superficial level, but a fuller analysis of the underlying neurophysiology shows that they in fact involve an active motor process. The obvious example would be maintained oculomotor fixation on a sacred object or point (e.g. the stigmata on a crucifix or on a particular part of an icon). Religious contemplation of such objects may appear to involve only stillness and quiet, but an analysis of the saccadic motor system shows that an active suppression of normal saccadic exploration is required. On the other hand, religious behaviours sometimes include actions that occur in the absence of voluntary awareness or perceived control (e.g. moving as a result of a divine power or being possessed by spirits). James referred to this last class of movements as ‘automatisms’ and linked them to hypnotic or subliminal suggestibility, as well as to ‘hysteria’.^3^ Finally, James gave other examples of religious practice as a tool that could modulate routine volitional motor control, including acts of endurance related to pilgrimage, or extraordinary control of respiration.

For us, James’ distinction can fruitfully be recast as a distinction between religious motor behaviour and religious experience of action. The first class of religious motor behaviour refers to actions that have special significance, but for which the experience of action itself is not altered by its religious use or meaning. This class includes many ritual behaviours and habitual motor patterns that occur in religious contexts, such as movements and postures associated with praying. For example, we assume that the experience of voluntary manual movement may be similar when someone makes the sign of the cross as a religious act and when the same person makes a physically comparable voluntary movement in another context, such as reaching for an object in their jacket pocket. The religious sign may have wider meaning for the person and may occur as part of a distinctive, wider experiential context. Importantly, however, the experience of the movement is not altered or transformed by this wider religious context. That is, the experience of movement itself may not be a religious ‘experience’: rather, it is a ‘normal’ movement that happens to have some religious ‘significance’ or association for the agent.

In other cases, in contrast, a distinctive or unusual experience of movement is intrinsically part of the religious experience itself. These cases are of special interest here because they imply an unusual relation between the physical facts of movement and the psychological facts of how the movement is experienced. Thus, actions made with the physiological voluntary motor system are normally experienced as self-caused, but clinical neurology shows that in some circumstances, the tight coupling between voluntary movement and experiences of volition and agency can come apart. Interestingly, scholarly discussions of religious experience generally focus on experiences of specific religious entities—e.g. thoughts of God—or on inner states and say little about how agents experience their own religious motor behaviours. Here we attempt to fill this gap by considering the experience of religious motor behaviours from the point of view of motor neuroscience. We will argue that a focus on the experience of action may help in understanding the logic and significance of religious motor behaviours, that the scientific concepts of motor systems neurology may contribute to this understanding, and that altered experience in some religious motor behaviours offers interesting parallels with some neurological symptoms.

## Religious rituals and motor behaviours

Ritualistic behaviours capture a wide range of motor phenomena that pervade the history of human societies.^4,5^ Ritualistic behaviours involve more automatic and stereotypic forms of motor output, habitual responses and composite sequences of voluntary actions that occur repeatedly and are associated with culturally relevant signifiers.^1,4,6^ As regards religious rituals in particular, many obvious examples include both linguistic and performative content, such as reciting of prayers, chanting and singing anthems. Importantly, the semantic content can be peripheral to the functions of these motor behaviours: many people recognize the experience of reciting a prayer without thinking, or even understanding, what the words mean. Such ritualistic behaviours may serve several different functions, including fostering social communication and cohesion, boosting motivation and goal commitment.^1,7,8^ Interestingly, ritualistic behaviour often increases in anxiogenic situations, suggesting that it may also play an important role in emotion regulation, particularly stress reduction.^9,10^ Accordingly, religious motor behaviours also increase during precarious times such as violent conflict^11,12^ and can have markedly anxiolytic effects.^13^ Ritualistic behaviour in general may also enhance the sense of control over novel or uncertain environmental contingencies.^14^

**Figure 1:**
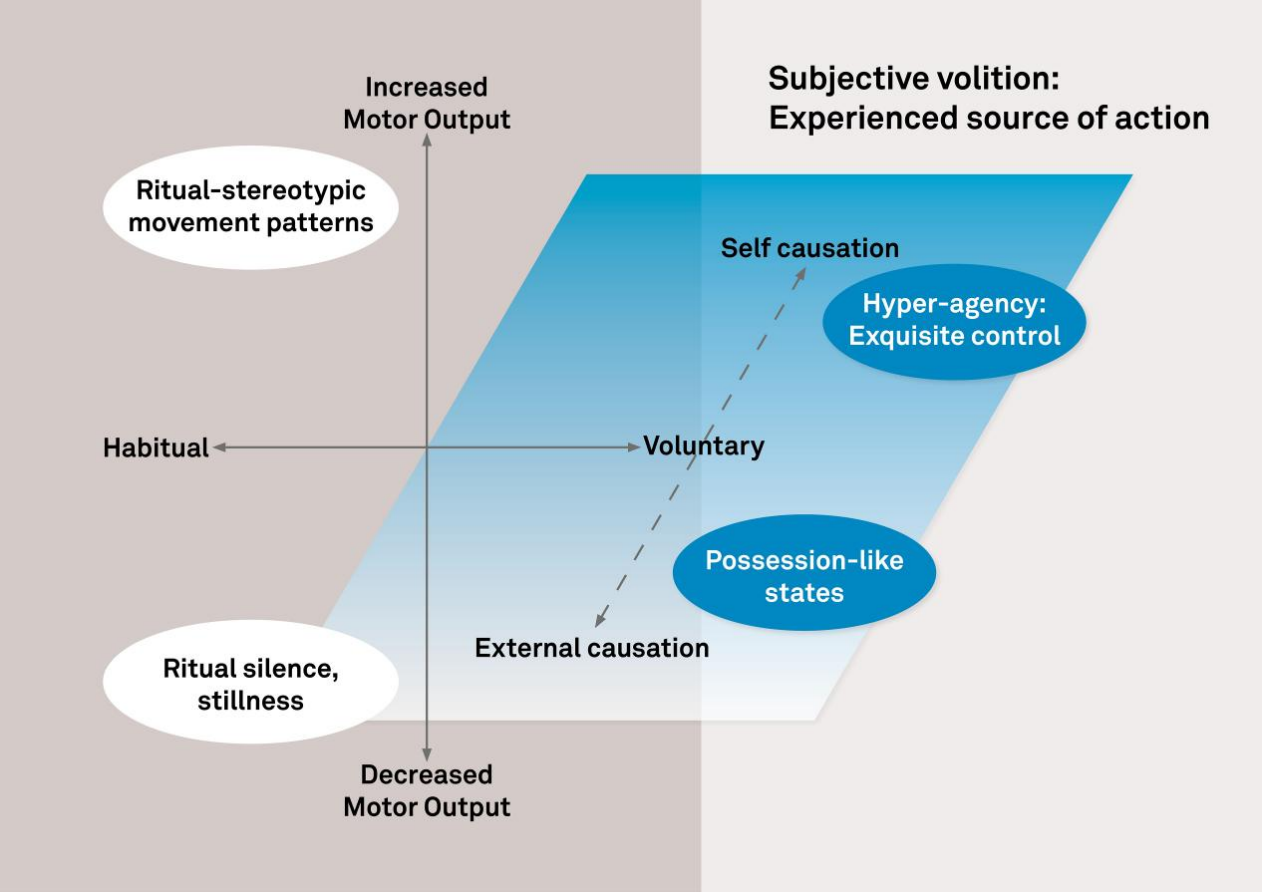
**A conceptual scheme for understanding religious behaviours from the perspectives of motor neuroscience.** Motor behaviours can be described by their positioning along two cardinal axes. First, movement varies in quantity from minimal to excessive (vertical axis). Second, the ‘controlling principle’ of movement also varies, since a given motor pattern may either reflect intense and deliberate cognitive control or may alternatively be habitual or automatic (horizontal axis). We propose that a crucial third dimension, characterizing how the agent experiences the ‘source’ of their own movement, is central to the experience of religious movement behaviours. An agent may experience a given movement as self-caused, and fully voluntary, or as guided by an external influence or cause. This third dimension explains how religious experience may include both possession-like states, in which the agent effectively loses the experience of volition, and also states of extraordinary voluntary control. Because this dimension describes the experience of ‘volition’, it is shown as an offset half-plane that applies more readily to voluntary movements than to habitual movements.

Several models have been put forward to explain the expression of ritualistic performance in humans. Some emphasize the high degree of cognitive control required for the execution of these behaviours (corresponding to the horizontal axis of Fig. 1) (e.g. Boyer and Lienard^15^). Respectively, detailed ritualistic action sequences, with their high fidelity of repetition, require attentional resources and cognitive effort. Thus, these rituals are not simply automatic or routine behaviours.^15^ On the other hand, there are clear parallels between habitual behaviours, religious rituals and compulsions.^9,16^ For example, entering a Christian orthodox church may trigger particular motor responses (performing the cross sign for three times and quickly reciting a specific prayer or parts of it), at least for some people. These context-specific motor habits may not involve the same level of continuous attention as some other actions generated by the voluntary motor system. Thus, the label of ‘religious rituals’ may in fact encompass a range of behaviours, which rely on different neurocognitive controlling mechanisms.

From a neuropsychiatric point of view, some ritualistic behaviours may also be described as compulsions. These are context-specific behaviours that may be the result of bodily sensations (e.g. urges, as in compulsive drug-taking^17^) or cognitions (i.e. obsessions, as in the example of obsessive-compulsive disorder^9^) where movements recur repetitively despite aversive consequences. Interestingly, some studies have suggested that highly religious people may experience obsessional symptoms related to scrupulousness, thought control, morality and cleanliness more often and/or more strongly than non-religious individuals.^18-20^ Given that the neurobiological basis of habits and rituals is also shared with the subcortical mechanisms responsible for compulsions,^9,16^ the exact boundaries between the different types of behaviours remain unclear. In fact, in many cases, it might be hard to discern the specific tipping point where repetitive religious rituals may shade into rigid obsessive-compulsive behaviours that approach clinical definitions of a disorder.

## Altered experience of volition during spiritual experience

The distinction between voluntary and involuntary motor phenomena can be made on either physiological or psychological grounds, as the axes of Fig. 1 show. The physiological voluntary motor system refers to activation of the cortical pathways controlling muscles. In contrast, psychological concepts of volition rely on the first-person experience of motivating, selecting, willing, initiating and controlling voluntary actions. One hallmark of volition is the conscious awareness of an intention to move. The distinctive subjective experience of volition allows people to discriminate movements they choose to make from body movements that just happen and so forms the basis for individual responsibility.^21^ Crucially, there are many phenomena where the physiological and psychological aspects of voluntariness dissociate.^22^ In particular, the class of motor phenomena often known as ‘automatisms’ generally involve activations of the cortical voluntary motor pathway without corresponding awareness of any intention to act. In the 19th century context of spiritualism, automatisms were interpreted as an external agent such as a spirit or deity, expressing itself through controlling an individual’s actions.^23^ Automatisms attracted a high level of popular attention in western societies at the time. Alternative, scientific explanations were proposed and tested.^24,25^ At the same time, psychologists and neurologists understood that automatism offered an interesting approach for exploring the structure of the nervous system and the control capacities of the human mind.^26,27^

**Figure 2:**
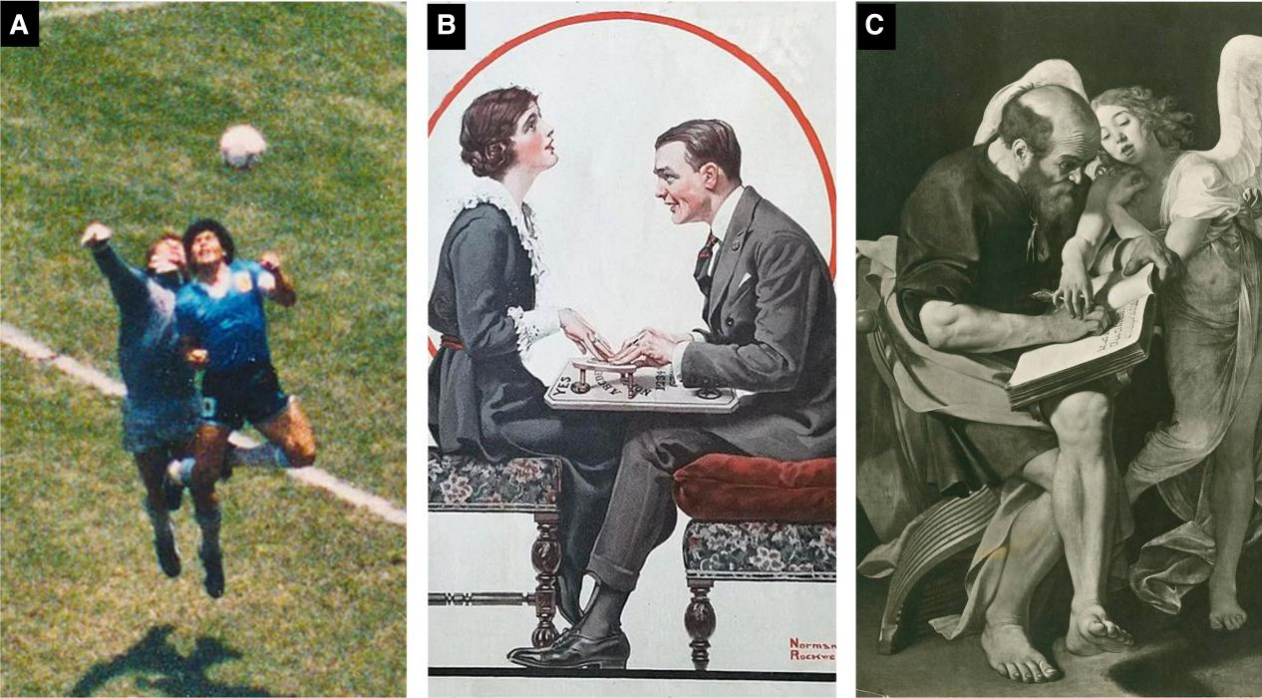
**Voluntary action attribution in religious motor behaviours.** Attribution of a voluntary action to an external cause can strongly influence the way the action is interpreted, framing both personal and societal understanding of the action. Examples of the importance of action attribution can be found in many areas of human life. (**A**) Upon scoring a goal that would have been disallowed at the Argentina versus England match in 1986, Maradona claimed this occurred ‘a little with the head of Maradona, and a little with the hand of God’. (**B**) A couple engaged in an Ouija board session, by Norman Rockwell. (**C**) Caravaggio’s painting of 1602, depicting Saint Matthew writing the Gospel under the guidance of an angel (images from: https://bettingamerica.com/maradona-hand-of-god-photo, © Joe O’Connell 2022/Licenced by Betting America; https://commons.wikimedia.org/wiki/File:Norman_Rockwell_Ouija_board_painting.jpg; https://en.wikipedia.org/wiki/File:Caravaggio_saint_matthew_DLI_06163009908.jpg).

Automatisms are defined as human actions that are not experienced as voluntarily controlled by the agent. An underlying difficulty of studying them is the unavoidable epistemological difference between first- and third-person perspectives. The action itself is an objective fact of bodily movement that can be observed by third parties, but there is no possibility for a third party to verify whether the agent experienced the movement as voluntary or involuntary. This means that automatisms are fertile ground for trickery and manipulation. In many settings, the agent may have a secondary reason to claim automatism (Fig. 2), and others may have reasons or willingness to believe the claim. In particular, ‘loss of control’ defences in law can potentially reduce severity of punishment.

Nevertheless, better-controlled experimental studies of suggestion and hypnosis confirm that, under some circumstances, agents can experience their own voluntary movements as if they were involuntary automatisms.^28^ In these experimental settings, the task demands of the situation can be controlled in a way that reduces secondary gain motivations to claim automatism. For example, Haggard *et al*.^28^ exploited the fact that the perceived time of onset for a voluntary keypress action occurs slightly earlier for active voluntary movements than for passive movements. When participants were suggested, via hypnosis, that they would move automatically, they perceived their voluntary movements to have onset times similar to passive movements.^28^ In this case, secondary reasons for claiming absence of volition seem unlikely, and, even if participants had such reasons, it is unclear how these reasons would influence judgements about movement onset times. These studies confirm that experiences akin to automatism can be elicited in many people.

‘Automatic writing’ is one particularly well-studied variety of automatism. It offers a striking example of voluntary movement without experience of volition and occurs in several religious and spiritual traditions. The terms psychography, passive or spirit writing^29^ have been used to describe the same basic phenomenon, and the use of devices such as the planchette and Ouija board share many similar features (Fig. 2). Religious traditions offer several examples of a divine entity guiding particular individuals in writing religious texts^30,31^ (Fig. 2). Artistic inspiration from Muses has been also thought to guide writers,^32^ suggesting a spiritual aspect to artistic creativity even in the absence of overt religious practice. The defining feature of automatic writing is that the agent does not have the experience of willing their hand to move—there is no experience of volition. There may be an experience of one’s hand moving, and of the writing taking place, but there is no feeling of willing the writing and therefore no sense of agency over what is written. Automatic writing is therefore a specific form of automatism and again reflects dissociation between action generation and experience of volition.^33^

There is a rich early history of scientific enquiry into the psychophysiological processes involved in automatic writing.^27,34^ During the late 19th century heyday of automatic writing, William Carpenter developed a theoretical framework of ‘ideo-motor action’ to describe how ideas may be directly translated into actions without conscious awareness of intending to act. Context and expectation of a particular outcome may be sufficient to facilitate execution of a motor programme even in the absence of an experience of volition or even against one’s reported will,^34^ a process which he termed ‘expectant attention’. In the example of the Chevreul pendulum,^35^ the expectation that the pendulum can or will swing seems to be sufficient for an agent to make the minimal movements required to set it swinging, even when they are explicitly instructed to hold it still and prevent any swinging motion. In this case, the voluntary motor pathway clearly produces small muscle contractions under visual control, but these are not experienced as voluntary acts.^23,34,35^ Two factors clearly contribute to the behaviour produced in this situation—the voluntary motor action of the agent, and the expectation and suggestion induced by the context. The important role of suggestion was also emphasized by Pierre Janet, writing around the same time.^36^

Interestingly, experimental analogues of automatic writing have been used in recent psychological studies to investigate conscious volition.^28,37-40^ Wegner *et al.*^23,37,41,42^ were able to reproduce several of the features of automatic writing and Ouija boards under controlled experimental conditions. On the one hand, these results confirm the power of suggestion, while on the other hand, they demonstrate that human experimental settings frequently include elements of suggestion even when suggestion is not made explicit. In his book *The Illusion of Conscious Will*, Wegner identified several specific cognitive mechanisms by which actions that are voluntary from a physiological point of view may nevertheless be perceived as involuntary.^23^ For example, if a minimal voluntary movement produces disproportionate outcomes, or results that are displaced (in the sense of being spatially and temporally remote from the action itself), or if the progress of action cannot be monitored in the normal way, an agent may attribute to an external cause an event that they have in fact caused themselves. In some cases, this misattribution can refer not just to action outcomes, but to the action itself—people may perceive as involuntary an action that is in fact voluntary.^41^

These phenomena are presumably facilitated by an intense preoccupation or desire to produce automatic behaviours, as well as by any general bias to attribute agency externally.^23^ The relation between sense of personal control and religiosity is complex.^43,44^ However, when agents cannot understand or cannot control their environment, they frequently attribute unexplained events to external agents, including religious deities and the supernatural.^45-48^ Interestingly, a recent study that examined the neural network associated with spirituality following structural brain lesions revealed overlaps with the brain networks associated with delusions and the alien/anarchic limb syndrome,^49^ in which patients experience a loss of voluntary control of a limb that compulsively responds to external stimuli. This overlap was suggested to reflect a commonality in the neural mechanisms underlying some religious beliefs and the experience of not being in control over one’s own motor behaviours, and points towards computations of agency and causal inference being key factors underlying spirituality.

## Complete loss of awareness of voluntary action and possession-like states

Above we have considered partial loss of experience of volition with respect to control of specific actions and have shown how this can be linked to those specific actions having religious meaning. In other cases, there can be a complete loss of experience of volition, where the agent has no experience of voluntary action at all, through an extended and generalized sequence of behaviour. Several areas of psychiatry and neurology highlight the possibility of complete loss of awareness of ongoing action. Functional neurological disorders provide prototypical examples where volition, action awareness and control are altered.^50^ Other relevant syndromes include gross delusions of control, dissociative identity disorder and amnesic (fugue) states.^51^ Equally, epileptology has documented loss of awareness during seizures, and sleep medicine has documented parasomnias, including somnambulism. However, in addition to these medical constructs, there is a well-documented and rich cultural history of behaviours that occur beyond voluntary control as part of religious experience and practice.^52^ Some religious practices aim to induce a specific mental state where self-awareness, memory and action control are retained (e.g. ‘trance states’), while others produce patterns of behaviour that may neither be experienced as willed nor even remembered, such as ‘possession trances’.^53^ Importantly, the first type of behaviour can occur as part of individual religious practices such as meditation; the second type typically takes place in a social context. The distinction between trance state and possession trance is not dichotomous, and indeed some religious practices involve elements of both.

**Figure 3:**
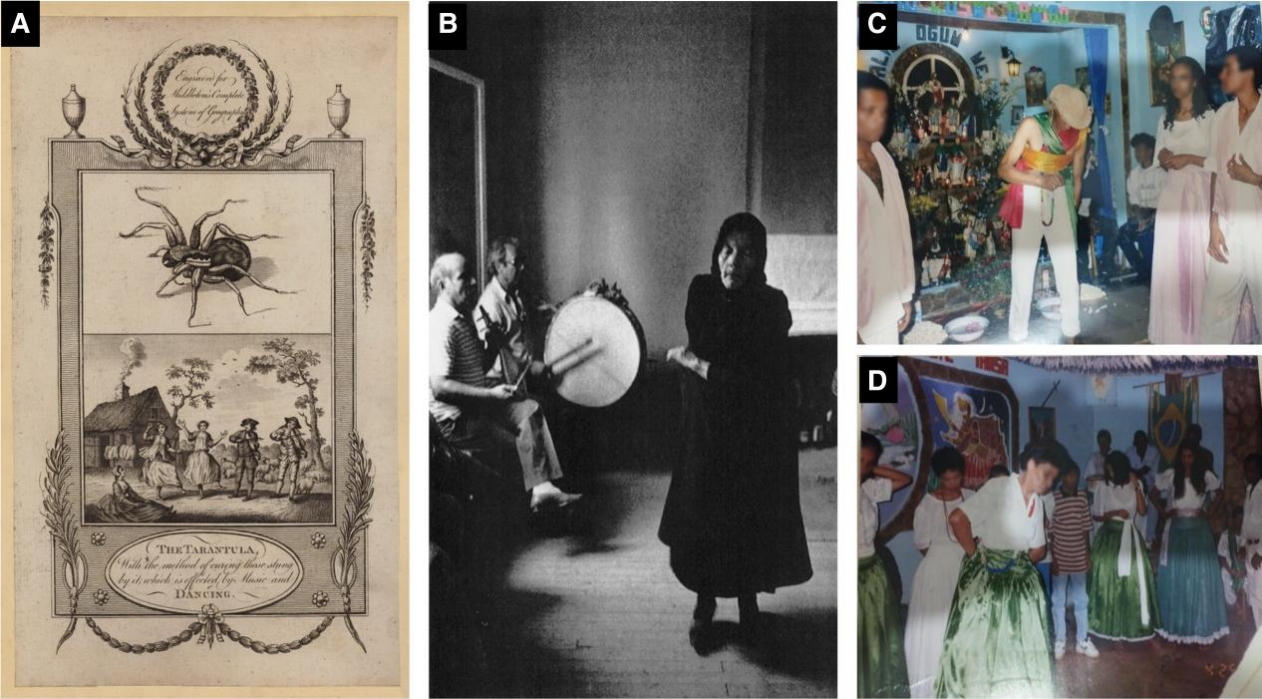
**Dance and religious motor behaviours.** Dancing is a distinctive religious motor behaviour and an important part of many religions. (**A**) Music and dance are both an expression of illness, and also a vehicle for healing, in examples such as tarantism. The text reads ‘The Tarantula, with the method of curing those stung by it, which is effected by Music and Dancing’ (illustration from Middleton^65^). (**B**) Ecstatic dancing to lyra and drum music in the Anastenaria fire-walking ritual (from Danforth^66^ reprinted with permission of Princeton University Press). (**C** and **D**) Ritualistic dancing and ceremonial spirit induction in Candomblé, Brazil. Both the ‘pai de santo’ (father of saints, equivalent to priest; upper image), and other participants (lower image) enter into trance states (reprinted with permission from the photographer Itamar Pereira de Aguiar’s personal collection).

States of altered action awareness, such as possession-like states, are often the result of a progressive induction phase. The induction phase may be facilitated by different factors, including the intake of psychotropic substances,^52,54^ and is often characterized by a common movement rhythm shared across a group of individuals. This may include bodily movements (dance), controlled breathing, chanting and music. Examples include Zar dance rituals, the Anastenaria, Sufi whirling, Voodoo drumming, Umbanda and Candomblé ceremonies, Pentecostal church rituals and many more.^52,55-57^ Historical events such as ‘the Dancing Plague’ or choreomania, tarantism, St. Vitus’ dance and tigretier may also have involved an element of ceremonial induction^58^ (Fig. 3). The spread of these states across several individuals within a group could depend on simple observation and imitation.^58,59^ However, some neuropsychologists have suggested a role for specialized cortical networks in interpersonal synchronization.^60-63^ Given its central role in religious practices, it is worth noting that music may be considered a special case of behavioural repetition and synchronization, facilitating social reward and widespread social bonding.^64^

From a phenomenological perspective, the types of behaviours encountered in possession-like states vary, but often include an increase in neuromotor activity with coarse movements such as body swaying, jerking, and shaking, as well as vocalizations, including ‘speaking in tongues’.^52,54,67^ Another type of behaviours involves a reduction of motor output, including immobility. Not uncommon are also episodes of sudden—or provoked—loss of postural control that lead to falling. Importantly, the motor phenotype may drastically change within a single possession-like episode. Descriptions of spirit possession and possession-like states may often involve sequences of motor agitation or convulsions alternating with periods of immobilization often as signs of a spiritual entity taking over the target host.^57^ In these states, different types of patterned behaviours and personality characteristics may also emerge. These are often attributed to the appearance of a new spiritual identity. The temporal duration of possession-like episodes may also differ, ranging from a few seconds and minutes to hours or even days and longer, depending on the contextual parameters of the induced state.^52,54,67^

The social organization of these practices also varies.^52,54,67^ One model involves a clear asymmetry of knowledge and power—the movements may be transmitted from a priest or similar authority figure to other participants. Alternatively, the priest may silence the movements, as in exorcism or similar. Another model is more symmetric—groups of people may participate in similar behaviours, either synchronously or asynchronously, without an obvious controller or leader.

## Psychological constructs and cognitive mechanisms of possession-like states

The phenomenon of spirit possession and possession-like states is multifaceted, highly varied culturally and strongly shaped by beliefs.^68^ Anthropological psychologists identified common themes that reoccur across such states, leading them to posit two theoretical constructs of psychological porosity and absorption.^69^ Porosity reflects the intuition that the boundary between the self and the world is permeable. It suggests that cognitions and affects may be the result of interactions that lie outside one’s typical realm of perception and action,^70^ as for example due to divine intervention.^69^ Porosity appears to be causally driven by culturally specific beliefs and therefore varies across societies.^69^ Absorption, on the other hand, describes the individual capacity for vivid immersion in sensory or imagined events.^69^ Absorption correlates with strong religious imagery and experiences, including perceiving the presence of God and other mystical-type experiences, as well as proneness to suggestibility and hallucinations.^71-73^ Importantly, porosity and absorption together determine the capacity to experience the workings of supernatural agents in the context of spiritual events.^69^ Interestingly, porosity and absorption^69^ may be altered either intentionally through participation in specific behaviours,^74^ or unintentionally through trauma.^75-77^

From a cognitive perspective, possession-like states have strong elements of visual learning, expectation and cultural transmission. The participants know the different behavioural routines involved in the practice from observation and often expect to participate in these behaviours themselves.^67^ In many cases, they may explicitly codify these behaviours and transmit them to others, as a specific or defining feature of the religious community.^67,78,79^ The strong involvement of learning and therefore expectation underlying such actions may include an element of self-suggestion. On this view, the agent’s religious motor behaviours could provide contextual cues that act as suggestions for a corresponding set of subjective experiences, including sensations, perceptions and beliefs. When the agent decides to initiate the religious motor behaviour, they may expect some specific religious experiences to follow.

## Parallels to functional neurological disorder

Functional neurological disorders that predominantly present with motor symptoms may phenotypically resemble some of the religious motor behaviours described above. Both categories of phenomena are often characterized by sudden state transitions or behavioural shifts between neurotypical and unusual movements. Critically, these changes in behaviour are also paralleled by changes in the subjective sense of agency. For example, there may be a transition between a state of full voluntary control (‘I decide and control what I do’) and a feeling of not being the author of one’s own actions (‘I can’t control these movements that are happening to me’). This transition may involve a single body part, as in cases of monomelic functional tremor or functional ‘alien limb’ reports. Alternatively, more than one body part may be affected, as in paroxysmal jerky movement disorders and non-epileptic seizures.^80-82^ These are alterations in the production and experience of movement. In principle, there might be similar alterations in the production and experience of stillness—absence of movement. However, these are less reported, perhaps because stillness is less salient than movement.

A recent meta-analysis showed increased levels of suggestibility in adults with functional neurological disorder—both measured using standardized behavioural scales and through direct symptom induction—compared to matched controls.^83^ Even though suggestibility might not explain all aspects associated with the occurrence of functional neurological disorders,^84^ it does provide insights into the structure of belief formation in these populations. Suggestibility is also thought to underlie the rapid occurrence of widespread functional symptoms that occasionally propagates through specific social groups and networks through the diffusion of strong affects, beliefs and expectations.^85,86^ Medical anthropological history provides plenty documented examples of such events, including the choreomanias described above,^59,87^ telephone sickness, the laughing mania and Havana syndrome.^88^ Other conditions, such as Morgellons disease,^89^ functional tics and tic-like behaviours^90,91^ as well as presentations resembling dissociative identity disorder,^92^ underscore the role of social media in the diffusion of information.^93^ How and why certain populations, including different age groups, are more susceptible to suggestion and the adoption of specific beliefs or behaviours remains poorly understood and warrants further investigation.^94^

Interestingly, in presentations of self-diagnosed ‘dissociative identity disorder’, the body serves as a host to ‘alters’ (other agents) that may take over different social roles and functions. This clinical presentation therefore has some similarities to spirit possession in the religious context.^92^ Similar parallels were drawn historically between rather ‘sudden’ possession-like states and seizures^95^ and even other paroxysmal motor phenomena, as also described above, including tics.^96,97^ For example, Charcot’s observations of religious artworks depicting possession led him to draw parallels between these states and functional disorders in his clinic, then labelled as ‘hysteria’.^98^ In the absence of direct evidence, it seems likely that epileptic and non-epileptic seizures were, or are still, often treated as cases of possession.^95^

How, and by whom, is the judgement made that an individual is possessed? In the culturally established cases described above, the social group typically has a consensus explanation of possession states. In other cases, individuals may volunteer the information that they have experienced possession, or they may retrospectively attribute their behaviours to a possession state. If a behaviour is culturally approved, a claim that is performed under possession is likely to be approved. Conversely, first-person claims that an inappropriate or sanctioned behaviour is a consequence of possession are typically dismissed and do not override social concepts of personal responsibility for action. Third-person judgements of possession are also common. They have been associated with ‘othering’ attitudes and hostility^95,99,100^ in some cases, but also with a special, or even elevated social status.^67,101^

## Religious beliefs, religious behaviours and faith healing

Actions with religious meanings and ritualistic behaviours have been deeply ingrained in the fabric of human evolution throughout history.^1,5,6^ We have already seen that ritualistic religious behaviours reduce anxiety while increasing the sense of control over one’s environmental contingencies.^10,27,102^ Moreover, they may foster personal growth and social identity, transmit knowledge, enhance creativity and self-expression, as well as promote cross-cultural communication and understanding.

The significance of many religious behaviours is often contextually driven. For example, some motor phenomena that may hold strong cultural and psychosocial meaning within the context of religion could equally be perceived as pathological symptoms of disorders within the medical framework.^103^ In events like the ‘kundalini awakening’, sudden motor and behavioural changes can be understood as part of a spiritual healing process, but have also been viewed as symptoms of functional neurological disorder, catatonia or even psychosis, depending on the type of presentation and the relevant context.^104,105^

Religious motor behaviours and medical symptoms often intersect, commonly driven by beliefs about illness and somatic symptoms.^106-109^ Religious motor behaviours may occur as part of understanding, interpreting or coping with symptoms of ill health or also to instigate trust and promote recovery. Therefore, knowledge about their expression and meaning can be crucial to facilitate effective communication and increase therapeutic compliance. This may be specifically relevant for symptoms such as those encountered in functional neurological disorder,^109,110^ as well as addictions and some impulse control disorders. In these latter cases, ‘surrendering to a higher power’ is a well-established therapeutic process. For example, recovery from alcohol and substance use disorders, or sexual compulsivity, can include agency off-loading in addition to behavioural rule-driven guidance as a specific element of the therapeutic programme.

## Conclusion

Religious rituals and actions are an integral part of human behaviour across societies. Their cultural significance transcends geographic borders and persists through time.^111^ Despite some suggestions that religions may be in decline,^112^ religious rituals, and specifically religious motor behaviours, continue to occupy a central role in the lives of many. From a movement neuroscience perspective, religious rituals and religious motor behaviours play a dual role. They regulate the type and amount of objective motor output while also shaping the agent’s subjective experiences. Highlighting the parallels between movement neuroscience and religious motor behaviours represents an opportunity for a new ‘way of seeing’ some clinical phenomena. Religious motor behaviours are a nexus of individual belief, social–cultural influences and ritual-stereotyped movement patterns. We suggest the same anchor points—belief, culture and ritual-stereotypy—are particularly important in understanding and influencing those movement disorders characterized by altered experiences of volition, both in neurology and psychiatry. To date, movement neuroscience has made rather few links to surrounding fields such as anthropology, sociology and religious studies. Among the advantages of pursuing such links could be a shift towards more culturally sensitive approaches to therapeutic interventions and an awareness of the diverse roles that culture plays in shaping motor behaviours and experiences.

## Data Availability

Data sharing is not applicable to this article as no new data were created or analysed in this study.
